# Thalamic abscess caused by a rare pathogen: *streptococcus constellatus*

**DOI:** 10.11604/pamj.2016.24.256.9468

**Published:** 2016-07-20

**Authors:** Özgür Şenol, Hikmet Turan Süslü, Necati Tatarlı, Mehmet Tiryaki, Bülent Güçlü

**Affiliations:** 1 Dr.Lütfi Kırdar Kartal Education and Research Hospital, Department of Neurosurgery, Ist anbul, Turkey

**Keywords:** Thalamic abscess, malnourishment, streptococcus constellatus, stereotactic aspiration

## Abstract

*Streptococcus constellatus* is a microorganism that lives commensally in the oropharyngeal region, urogenital region, and intestinal tract. However, it can cause infection in patients with certain predisposing factors. Rarely, this microorganism can cause a brain abscess. Thalamic localization of brain abscesses is much rarer than abscesses in other locations of the brain. Brain abscess caused by *streptococcus constellatus* are very rarely been reported in the literature. We present a rare case of a left-sided thalamic abscess caused by *streptococcus constellatus* in a 25-year-old male patient who was injured by shrapnel pieces in the head and who was malnourished. The patient was successfully treated by stereotactic aspiration and antibiotherapy.

## Introduction

*Streptococcus constellatus* is a member of the Streptococcus family and is part of the normal flora of the oral cavity, urogenital region, and intestinal tract. It can cause purulent infections in patients with cirrhosis, diabetes, malignancy, malnourishment, or conditions that cause immunosuppression [1]. Brain abscesses caused by *streptococcus constellatus* are reported very rarely in the literature [2, 3]. Thalamic abscesses are much rarer than abscesses in other locations of the brain [4, 5]. In the literature, there is no single thalamic abscess caused by *streptococcus constellatus*. We present a rare case of a left-sided thalamic abscess that was caused by *streptococcus constellatus* in a 25-year-old malnourished male patient who was wounded in the head by shrapnel. The patient was successfully treated with stereotactic aspiration and antibiotherapy.

## Patient and observation

**Figure 1:**
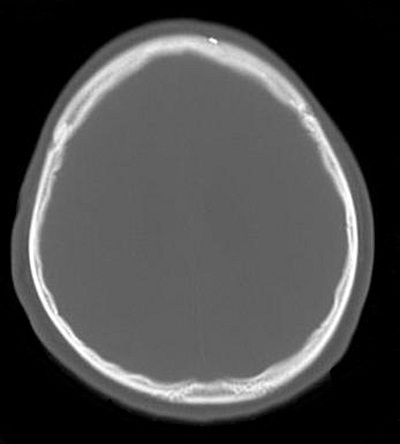
Bone window of cranial CT scan showing shrapnel in left frontal bone (same side as thalamic abscess)

**Figure 2:**
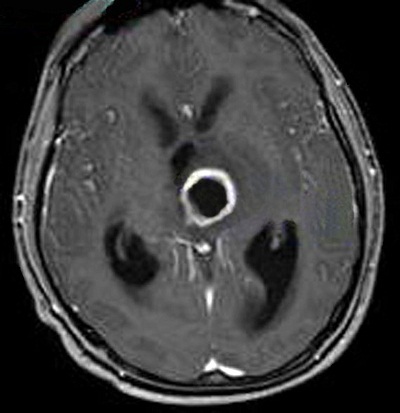
T1-weighted axial MR image with contrast showing a thalamic ring-enhancing lesion in the left side of the brain

**Figure 3:**
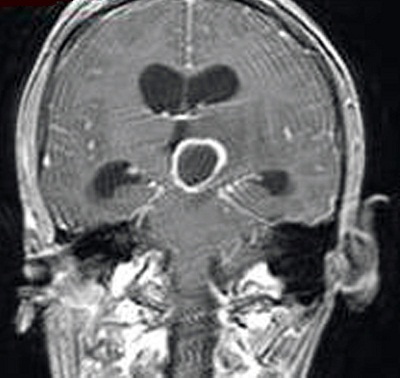
T1-weighted coronal MR image with contrast of the same patient

**Figure 4:**
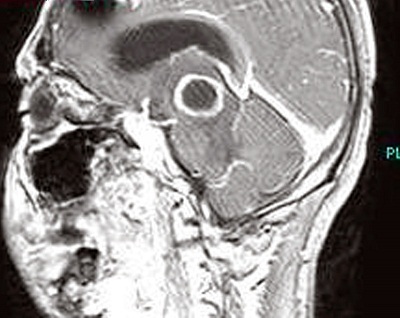
T1-weighted sagittal MR image with contrast showing a thalamic ring-enhancing lesion and artifact due to shrapnel in left frontal bone

**Figure 5:**
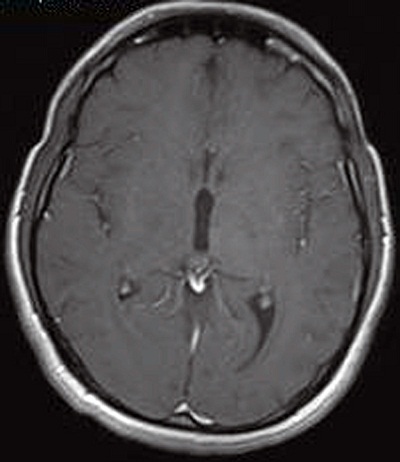
T1-weighted axial MR image with contrast four months after CT-guided stereotactic biopsy and antibiotic therapy, showing a substantially reduced abscess volume and a residual encapsulated area

**Figure 6:**
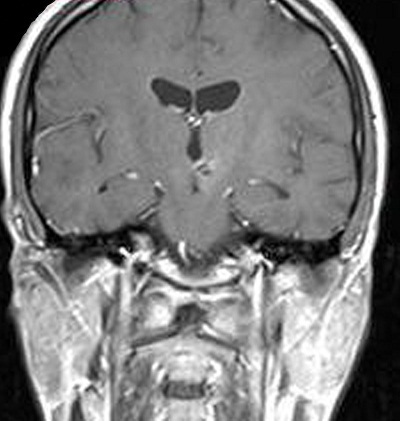
T1-weighted coronal MR image with contrast of the same patient

A 25-year-old male was brought to the hospital with a chief complaint of headache associated with vomiting, mild fever, and right-sided paresis. He had been found near a small river, lying on the ground in very bad condition. His history revealed that he had been wounded in the head by pieces of shrapnel during a civil war in a different country. He had escaped from the war and had not been sufficiently nourished for approximately a month. The patient had no previous history of alcohol abuse, dental infection, or any other debilitating disease. Physical examination revealed a small scar from the healed wound in the left frontal area. Neurological examination showed that the patient was lethargic and had right-sided hemiparesis. Bone windows on computed tomography (CT) revealed small shrapnel pieces in left frontal bone. Gadolinium-enhanced magnetic resonance imaging (MRI) of the cranium showed a mass lesion measuring 24 x 26 x 22 mm in the region of the left thalamus. There was marked ring enhancement, perilesional edema, and a slightly deviated third ventricle, but no enlargement of the ventricular system (Figure 1, Figure 2, Figure 3 and Figure 4). A CT-guided stereotactic biopsy was performed of the lesion and 15 ml of yellowish-brown purulent material was aspirated. Culture of the pus showed the presence of *streptococcus constellatus*. The *s. constellatus* was susceptible to meropenem and vancomycin, and appropriate antibiotherapy was started. By the end of the second postoperative week, all symptoms had resolved. Follow-up cranial MRI after 12 weeks of the intravenous antibiotic regimen showed normalization of abnormal lesions and disappearance of the remaining brain abscess (Figure 5 and Figure 6).

## Discussion

Thalamic localization of brain abscesses is uncommon [6]. In most cases, underlying sources of infection are found, such as congenital heart disease, intrathoracic and abdominal infection-based sepsis, dental caries, otitis media, or sinusitis [6]. However, in some cases no source of sepsis or any predisposing factors are found. The most common organisms in the reported thalamic abscesses are Streptococci and anaerobes. However, the incidence of culture-negative cases accounts for 28 percent of reported thalamic abscesses [6].

*streptococcus constellatus* is a member of the normal flora of the mouth, gastrointestinal tract, and genitourinary tract and is often associated with purulent infections. Brain abscess caused by *streptococcus constellatus* is very rare [2, 3, 6, 7]. In all reported cases of *streptococcus constellatus* abscess, there was either underlying pathology or a history of immunosuppression [2, 3, 6, 7]. Our case is the first reported case in the literature of a single thalamic abscess caused by *streptococcus constellatus*. In our case, there is a history of malnutrition and a shrapnel wound in the same side of the head as the thalamic abscess. The history of malnutrition and malnutrition-related immunosuppression was the probable underlying condition related to this cerebral abscess; however, we are not sure about the relationship between the shrapnel wound and the thalamic abscess on the same side of the head because the shrapnel pieces were located subcutaneously over the frontal bone and did not enter either the bone or the brain parenchyma.

The best surgical management of thalamic abscesses remains controversial. One of the important goals of thalamic abscess surgery is to prevent intraventricular rupture of the abscess in the operation. Treatment options include stereotactic aspiration, free-hand aspiration through a burr hole, stereo-endoscopic aspiration, ultrasound-guided aspiration, surgical transventricular approach, and medical management [5, 7, 8]. Stereotactic aspiration remains the preferred treatment as it drains the contents of the abscess, reduces mass effect, carries less risk of intraventricular rupture preoperatively, and confirms diagnosis. In our case, the abscess was drained by CT-guided stereotactic technique. After confirmation of the exact cause, appropriate antibiotic therapy healed the patient with no radiological or neurological sequelae.

## Conclusion

Thalamic abscesses are much rarer than abscesses in other locations of the brain. In the literature, there is no single thalamic abscess caused by Streptococcus constellatus. It may be taken into consideration and is critical that pyhsicians include this condition in differential diagnosis.
